# Supplementary figures and images for: Modeling spatial evolution of multi-drug resistance under drug environmental gradients
Source: PLoS Comput Biol. 2024 May 31;20(5):e1012098. doi: 10.1371/journal.pcbi.1012098 (PMC11142541; doi:10.1371/journal.pcbi.1012098)

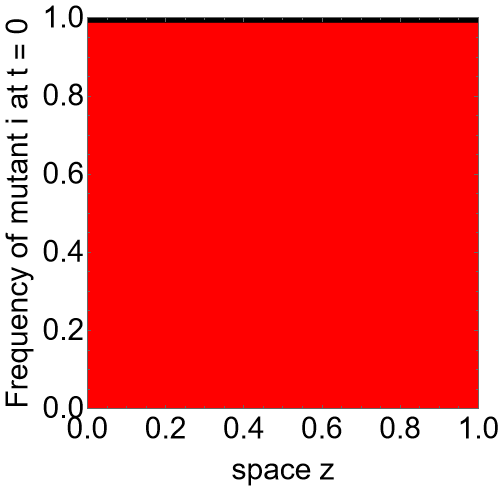

Supplement: S1 Video — (GIF) [file pcbi.1012098.s002.gif]

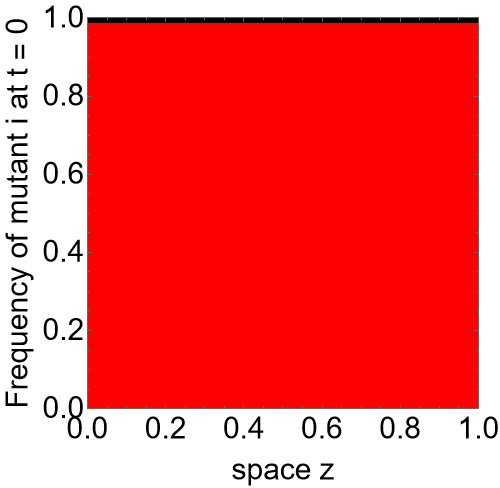

Supplement: S2 Video — (GIF) [file pcbi.1012098.s003.gif]
